# Estrogens and Estrogen Receptor Modulators in Cancer Research and Therapy

**DOI:** 10.3390/cancers15174318

**Published:** 2023-08-29

**Authors:** Oliver Treeck

**Affiliations:** Department of Gynecology and Obstetrics I, University Medical Center Regensburg, 93053 Regensburg, Germany; otreeck@csj.de

Estrogens affect oncogenesis and tumor progression in a variety of cancer entities. In breast cancer, endocrine therapy (ET), including selective estrogen receptor modulator (SERM) tamoxifen, aromatase inhibitors (AIs) and selective estrogen receptor degraders (SERDs), has been proven to block the tumor-promoting action of this steroid hormone, which is primarily mediated by estrogen receptor (ER) α. However, resistance to endocrine breast cancer therapy remains a major clinical problem. ERβ, in contrast, is reported to act as a tumor suppressor in various cancer types such as breast and prostate cancers, whereas the G-protein-coupled estrogen receptor GPER1 seems to have pleiotropic roles in cancer progression. In striking contrast to cancer entities that have been early recognized to be adversely affected by the tumor-promoting effects of estrogens, other cancer types that are not classically considered to be hormone-dependent, e.g., lymphomas, interestingly show a lower incidence and a better prognosis in females when compared to males. Although the reason for this sex-specific difference is not fully understood, the fact that particularly pre-menopausal women but not post-menopausal women have a lower lymphoma incidence and a longer survival raised the question, to what extent estrogens might exert protective effects in this cancer entity. In this Special Issue, a study by Huang et al. intended to elucidate the mechanisms underlying the increased male incidence of developing diffuse large B-cell lymphoma (DLBCL) and their adverse prognosis [1]. In this study, based on the gene expression data from a publicly available cohort of 746 DLBCL samples, the authors found 1293 differentially expressed genes (DEGs) when comparing males and females, and identified 345 activated B-cell lymphoma (ABC) DLBCL DEGs when comparing post- and pre-menopausal women. When combining the DEGs in females vs. males and the DEGs in pre- vs. post-menopausal females, nine putative estrogen-regulated genes in ABC DLBCL were identified. Among them was the up-regulated gene NR4A2, which was previously reported as a tumor suppressor in lymphoma and was shown to be associated with better survival in ABC DLBCL females. The downregulated gene MUC5B was reported to be associated with an adverse phenotype in several cancers. Importantly, NR4A2 and MUC5B were confirmed to be regulated by estrogens in an opposite manner when the ABC cell line U2932 was grafted in mice. This study demonstrated the sex-dependent differences and the female-reproductive-age-dependent differences in gene expression in DLBCL and identified genes that might be involved in estrogen-dependent mechanisms underlying the lower DLBCL incidence and better prognosis of women.

With regard to estrogen-dependent ERα-positive breast cancer, due to the limitations of endocrine therapy regimens emerging from ET resistance, many efforts are currently being made to overcome this major clinical problem. On the one hand, gaining a deeper understanding of the molecular mechanisms leading to ET resistance is expected to reveal novel predictive markers for therapy efficacy, which could also be used for therapy monitoring. On the other hand, the identification of molecules or pathways affecting ET response might open up novel strategies to increase therapy efficacy and might lead to a rationale for the combination of ET drugs with novel targeted drugs. In this Special Issue, an original report by Natori et al. found that the marker NEDD4 (neural precursor cell expressed developmentally downregulated 4–1) decreases ET efficacy [2]. The authors examined the prognostic value of E3 ligase NEDD4, which is known to degrade proteins, including ERα, in breast cancer patients receiving endocrine therapy. In a retrospective cohort study enrolling 143 patients with hormone-receptor-positive, HER2-negative early breast cancer, who had received neoadjuvant/adjuvant hormone therapy, both disease-free survival and overall survival were shown to be significantly longer in the group with a low NEDD4 expression than in the group with a high NEDD4 expression (*p* = 0.048 and *p* = 0.022, respectively). In the in vitro part of this study, a knockdown of NEDD4 in breast cancer cell lines was confirmed to elevate ERα protein levels and to increase sensitivity to tamoxifen treatment. The presented data suggest that NEDD4 might be a promising marker to predict the efficacy of therapy regimens that are based on blocking the tumor-promoting, ERα-mediated action of estrogens. Currently, many efforts are being made to increase the efficacy of AIs in breast cancer therapy using a combination with other drugs. In the in vitro study by Ferreira-Almeida et al., the addition of cannabidiol (CBD), particularly to the steroidal AI exemestane, increased its growth inhibitory and apoptotic effects on ERα-positive MCF-7 breast cancer cells and was shown to block ERα and ERK1/2 activation [3]. This study opens up a new potential promising line of research for the improvement of adjuvant ERα-positive breast cancer therapy, particularly for patients undergoing exemestane treatment.

To further engage the problem of de novo or acquired resistance to endocrine breast cancer therapy, a deeper understanding of its mechanisms is required, which can lead to the identification of novel predictive markers and therapy targets. This Special Issue contains a review article focusing on non-coding RNAs (ncRNAs), which regulate estrogen signaling and are thereby able to affect ET efficacy [4]. A surprisingly high number of 50 micro RNAs (miRNAs), but also, to a lesser extent, long non-coding RNAs (lncRNAs) and circular RNAs (circRNAs), were identified in vitro, which target and downregulate the *ESR1* gene or other components of estrogen signaling. Several miRNAs downregulating *ESR1* expression were demonstrated to promote cellular resistance to tamoxifen, fulvestrant or Ais, whereas other miRNAs reduced the ET response of breast cancer cells by targeting the ERα co-activators *FOS*, *MED1*, *FOXO3*, *GATA3* and *NCOA3*, or estrogen effectors like *CCND1* and *CDKN1B*. Some miRNAs were reported to enhance tamoxifen response, e.g., by targeting ERα co-repressors. Furthermore, several lncRNAs and circRNAs were identified that interfere with genes involved in estrogen response via different mechanisms, either enhancing or counteracting ET resistance. The in vitro identification of ncRNAs affecting the efficacy of ET drugs on BC cells led to the implementation of clinical studies correlating the serum levels of such ncRNAs with patients’ ET responses, which provided proof of principle, e.g., demonstrating high levels of miR-221 or lncRNA HOTAIR to be associated with tamoxifen resistance in BC patients. It is expected that the quantification of certain ncRNAs in liquid biopsies will provide a versatile non-invasive method to predict or monitor ET response. On the other hand, targeting ncRNAs via different strategies is an interesting approach to counteract ET resistance.

Finally, a comprehensive review article focused on the role of the non-classical estrogen receptors ERβ and GPER1 and estrogen-related receptors in endometrial cancer (EC) and ovarian cancer (OC) [5]. The reviewed studies clearly suggest the tumor suppressor role of ERβ that was previously reported in breast and prostate cancers to also be present in ovarian cancer, as was demonstrated by a variety of in vitro studies, including ones employing ERβ modulators, and by reports showing ERβ to be associated with the improved survival of OC patients. Although in vitro studies also suggested a tumor-suppressing role of ERβ in EC, studies based on ERβ expression in EC tissues had inconsistent results, indicating the need for further investigation. The G-protein-coupled estrogen receptor GPER1 was reported to exert tumor-promoting as well as tumor-suppressing actions both in OC and EC. These contradictory data, which might emerge from the pleiotropic functions of GPER1, show the need for further in-depth studies to clarify the role of GPER1 in these cancer types, also addressing its subcellular localization. With regard to estrogen-related receptors (ERRs), the available data suggest that these orphan receptors, particularly ERRα, have tumor-promoting roles in OC and EC. However, the authors conclude that further studies including larger patients’ collectives are necessary to further elucidate the roles of ERRs in these cancer entities.

In conclusion, the further investigation of estrogen actions in different cancer entities remains a vital challenge, as does the major clinical problem of resistance to ERα modulators in BC therapy. The presence of estrogen effects in cancer types that are not classically considered to be estrogen-dependent, which might be mediated by receptors like ERβ and GPER1, as well as the emerging role of ncRNAs in estrogen signaling, open up promising avenues of research with the potential to further improve cancer therapy.

## References

[1] Huang D., Berglund M., Damdimopoulos A., Antonson P., Lindskog C., Enblad G., Amini R.-M., Okret S. (2023). Sex- and Female Age-Dependent Differences in Gene Expression in Diffuse Large B-Cell Lymphoma—Possible Estrogen Effects. Cancers.

[2] Natori Y., Suga J., Tokuda E., Tachibana K., Imai J.-I., Honma R., Azami Y., Noda M., Sasaki E., Watanabe S. (2023). E3 Ubiquitin Ligase NEDD4 Affects Estrogen Receptor α Expression and the Prognosis of Patients with Hormone Receptor-Positive Breast Cancer. Cancers.

[3] Almeida C.F., Teixeira N., Valente M.J., Vinggaard A.M., Correia-da-Silva G., Amaral C. (2023). Cannabidiol as a Promising Adjuvant Therapy for Estrogen Receptor-Positive Breast Tumors: Unveiling Its Benefits with Aromatase Inhibitors. Cancers.

[4] Treeck O., Haerteis S., Ortmann O. (2023). Non-Coding RNAs Modulating Estrogen Signaling and Response to Endocrine Therapy in Breast Cancer. Cancers.

[5] Schüler-Toprak S., Skrzypczak M., Gründker C., Ortmann O., Treeck O. (2023). Role of Estrogen Receptor β, G-Protein Coupled Estrogen Receptor and Estrogen-Related Receptors in Endometrial and Ovarian Cancer. Cancers.

